# Tuberculosis, the Disrupted Immune-Endocrine Response and the Potential Thymic Repercussion As a Contributing Factor to Disease Physiopathology

**DOI:** 10.3389/fendo.2018.00214

**Published:** 2018-05-01

**Authors:** Luciano D’Attilio, Natalia Santucci, Bettina Bongiovanni, María L. Bay, Oscar Bottasso

**Affiliations:** Instituto de Inmunología Clínica y Experimental de Rosario, UNR-CONICET, Rosario, Argentina

**Keywords:** tuberculosis, immune-endocrine communication, inflammation, thymic involution, pathophysiology, hormones

## Abstract

Upon the pathogen encounter, the host seeks to ensure an adequate inflammatory reaction to combat infection but at the same time tries to prevent collateral damage, through several regulatory mechanisms, like an endocrine response involving the production of adrenal steroid hormones. Our studies show that active tuberculosis (TB) patients present an immune-endocrine imbalance characterized by an impaired cellular immunity together with increased plasma levels of cortisol, pro-inflammatory cytokines, and decreased amounts of dehydroepiandrosterone. Studies in patients undergoing specific treatment revealed that cortisol levels remained increased even after several months of initiating therapy. In addition to the well-known metabolic and immunological effects, glucocorticoids are involved in thymic cortical depletion with immature thymocytes being quite sensitive to such an effect. The thymus is a central lymphoid organ supporting thymocyte T-cell development, i.e., lineage commitment, selection events and thymic emigration. While thymic TB is an infrequent manifestation of the disease, several pieces of experimental and clinical evidence point out that the thymus can be infected by mycobacteria. Beyond this, the thymic microenvironment during TB may be also altered because of the immune-hormonal alterations. The thymus may be then an additional target of organ involvement further contributing to a deficient control of infection and disease immunopathology.

## Tuberculosis (TB) and its Main Pathophysiological Features

*Mycobacterium tuberculosis* (*M. tuberculosis*), the etiologic agent of TB, is responsible for more deaths worldwide than any single pathogen with an estimated 10.4 million patients and 1.3 million deaths, annually in 2016 (1). Most cases of primary TB infection are clinically and radiologically unapparent. These individuals remain persistently infected by *M. tuberculosis* constituting non-contagious carriers of the bacillus but setting the stage for subsequent reappearance. About 5% of patients pass from latency to post-primary disease within 2 years of primary infection and another 5% do so in later lives. While most cases of post-primary TB in immunocompetent adults arise from reactivation from latent infection, molecular studies showed that exogenous reinfection accounts for a significant percentage of cases in some areas of the world. Adult post-primary TB typically affects the best aerated lung regions, preferably the upper lobes (2, 3). The histopathological hallmark is a granuloma composed of epithelioid cells with variable numbers of Langhans’ giant cells surrounded by lymphocytes and a central zone of caseation necrosis and variable degree of fibrosis (3–6). The structure is surrounded by a fibrous capsule which constitutes a contention barrier. A spectrum of lesions may be seen from a hard granuloma without necrosis and rare organisms to the one with multibacillary necrotic lesions in the central zone, even within the same patient (7, 8).

Human infection with *M. tuberculosis* can result in a varied degree of organic compromise, ranging from an asymptomatic process to frank lung pathology with cavity formation and high bacillary load. Such clinical spectrum relies on a complex series of interactions between *M. tuberculosis* and the host immune response (4). The defensive reactions mainly involve the microbicidal effect of activated macrophages and the capacity of cytotoxic lymphocytes to destroy infected macrophages. Upon phagocytosis macrophages can produce or receive the influence of different cytokines rendering them more effective in suppressing bacillary replication and possibly destruction of the *mycobacterium*, i.e., IFN-γ (4, 9). This cytokine is secreted primarily by T lymphocytes, particularly the so-called Th1 cells which are involved in the protective immunity toward the mycobacteria (2), although in some circumstances Th1 immunity can also result in unbalanced pulmonary inflammation (9). Possibly, a better correlate of protection deals with the profile of cytokine production, since patients with TB disease showed elevated frequencies of *M. tuberculosis*-specific CD4 T cells expressing only TNF-α or TNF-α^+^IFN-γ^+^CD4^+^ T cells, whereas cases with latent TB infection showed greater frequencies of polyfunctional TNFα^+^IFN-γ^+^IL-2^+^ *M. tuberculosis* specific CD4^+^ T cells (10–12).

In our laboratory, we have shown that patients with mild forms of TB have a suitable Th1 response pattern and that it is gradually reduced as the disease progresses (13, 14).

The other mechanism involved in protection comprises the elimination of infected macrophages by cytotoxic lymphocytes through the classical events of granules containing perforin and granzymes or the induction of apoptosis through the Fas-FasL interaction. Following the formation of apoptotic bodies, they are ingested by phagocytes *via* the efferocytosis. The efferosome surrounds the newly incorporated apoptotic cell followed by successive events of fusion with lysosomes, delivery of hydrolytic enzymes to this efferosome in maturation and gradual increase of its acidification to finally proceed with the destruction of apoptotic cells (15). Nevertheless, an increased apoptosis may sometimes spread the infection to neighboring macrophages considering the extensive apoptosis seen within caseating granulomas of patients with lung TB (16).

## The Altered Immune-Endocrine Communication in TB

Tuberculosis constitutes a natural model wherein the essential processes required for mounting successful defensive strategies and homeostasis maintenance may result detrimental when the infection becomes chronic, as the accompanying inflammation. Our studies point out that such disorder not only affects the containment mechanisms but also the immune-endocrine communication, favoring a more morbid disease course (17).

The bidirectional communication between the neuroendocrine and immune systems is well-known. While products from the immune response can modify the functioning of the endocrine system, hormones like adrenal steroids directly affect the activity of immune cells and hence the course of disease-states with an inflammatory, autoimmune, or infectious background. This interconnection between the immune and the neuroendocrine systems is partly due to the stimulatory activity of inflammatory cytokines on the hypothalamus pituitary adrenal (HPA) axis. Briefly, cytokines such as IL-6, IL-1β, and TNF-α stimulate the production of corticotropin-releasing hormone (CRH) in the hypothalamus with subsequent release of adrenocorticotrophin into the pituitary gland, which in turn promotes the secretion of steroid hormones at the level of the adrenal cortex: cortisol and dehydroepiandrosterone (DHEA) (18, 19). Both hormones are known to exert relevant immunomodulatory effects. For instance, glucocorticoids (GCs) can inhibit Th1 responses, whereas their natural antagonist DHEA is able to favor them (18, 19). As part of integrated physiological circuits, these endocrine reactions, particularly the HPA axis, represent a well-conserved mechanism to control/support an intense immune-inflammatory reaction as well as for the early mobilization of immune cells and their redistribution to mount an adequate defensive response. Nevertheless, when the inflammatory condition becomes persistent such prolonged immuno-inflammatory aggression leads to a misuse of these evolutionarily conserved control mechanisms contributing to exacerbate host damage (20, 21).

Regrettably, the implication of these reciprocities in the field of pathogenesis, prognosis and treatment of chronic infectious diseases remains underestimated.

Beyond inhibiting the development Th1 cells in favor of Th2 responses (22, 23), GCs also interfere with gene expression for pro-inflammatory cytokines, by hindering nuclear factor kappa B (NF-κB) signaling (24, 25). More recent studies reveal that during the immune response GCs exert differential effects on effector and regulatory T cells with an intense inhibition in the proliferation of the former and a differential apoptosis of the latter (26). Under certain conditions, GCs may also have pro-inflammatory effects by some not well characterized mechanism. These apparently opposing actions would work together to prepare the immune system to respond to the stressful stimulus (pro-inflammatory effect) and subsequently to restore homeostasis—an anti-inflammatory effect—which is obviously the most prominent role of GCs (27). On its own, DHEA is also able to inhibit the secretion of pro-inflammatory cytokines such as IL-6 and TNF-α (28, 29).

To ascertain the immunoendocrine alterations during TB, we initially studied the circulating levels of cytokines and hormones such as IFN-γ, IL-10, IL-6, cortisol, DHEA, GH in male TB patients with different degrees of lung involvement and free from endocrine disorders, or treatment with corticosteroids or immunomodulatory drugs. Patients presented increased levels of IL-6, IFN-γ, and cortisol, whereas DHEA levels were well below the control values, the lowest levels corresponding to those with advanced disease (30). In line with this, other studies in active TB patients from Turkey and South Africa also revealed decreased DHEA levels (31–33), whereas cortisol concentrations appeared unchanged (31, 32) or slightly increased (33).

At the *in vitro* level, treatment of peripheral blood mononuclear cells (PBMCs) with cortisol, at slightly supraphysiological levels, resulted in a decreased proliferation and production of IFN-γ to mycobacterial antigen stimulation, with no changes in IL-10 production (34). DHEA, on its own, caused a significant decrease in the production of TGF-β by PBMCs of patients with advanced TB (34), a cytokine which is well known for its suppressive and harmful effects on TB (17). When studying the functional capacity of dendritic cells exposed to *M. tuberculosis* antigens, cortisol significantly inhibited the secretion of IL-12, IFN-γ, and IL-10 by these cells, whereas DHEA increased the expression of MHC-I, MHC-II, and CD86, in addition to improving IL-12 production and decreasing IL-10 secretion (35). DHEA also inhibited the intra-macrophage bacillary growth, which was related to a higher level of autophagy (36). Collectively, our studies are consistent with the view of a respective detrimental or favorable influence of cortisol and DHEA on the anti-TB immune response.

As part of this interrelation between the endocrine system and the immune system, culture supernatants of PBMCs from TB patients stimulated with mycobacterial antigens inhibited the secretion of DHEA by the human adrenal cell line NCI-H295-R (30) whereas treatment with anti-TGF-β neutralizing antibodies reversed this inhibitory effect (37). This observation reinforces the close network of influences underlying immuno-endocrine regulation, particularly the production level of adrenal steroids and immune mediators.

Changes in the immune-endocrine communication may be also implicated in situations further contributing to disease morbidity. In fact, we have demonstrated that the defective *in vitro* immune responses of TB patients to mycobacterial antigens was related to their reduced body mass index (BMI), which was negatively correlated with IL-6 circulating levels (38). This cytokine is known to play a role in the regulation of lipid metabolism and studies in TB patients indicate that increased IL-6 concentrations were associated with loss of appetite (39). Regarding hormones, GCs may favor a loss of body mass since they mobilize lipid stores by inducing lipolysis in fat cells *via* stimulation of a hormone-sensitive lipoprotein lipase. Also, GCs inhibit protein synthesis and stimulate proteolysis in muscle cells (40), in addition to reducing food intake and inducing body weight loss, probably *via* increased hypothalamic CRH levels, which seems to be catabolic (41). In essence, the immune-endocrine profile is adverse for the patient being involved in the reduction of body weight or consumption state during infections. This situation, defined as cachexia is a multifaceted metabolic disturbance present in several chronic inflammatory diseases or end stage neoplasms comprising weight loss, adipose tissue and skeletal muscle depletion, along with reduced appetite. The mechanisms underlying cachexia development are complex, encompassing the participation of neurologic, metabolic, immunologic, and endocrinological factors (42–44). In this context, we have recently found that the lower BMI of patients coexists with reduced levels of leptin, whereas concentrations of IL-6, cortisol, IL-1β, and adiponectin were increased (45).

The basis for the above described alterations has to do with the acute phase response (46), an adaptive reaction trying to be beneficial for the host at least during the early infection (46). This leads to a new metabolic set point attempting to attain an optimal functioning of the immunological needs, without affecting requirements of some often-competing physiological functions (47, 48). Since energy is not a limitless resource, when the infection becomes chronic metabolic deficit establishes further affecting the defensive reaction and disease outcome.

The link between energy supply and the immune response is supported from a study carried out in Africa in which the metabolic needs to cope with measles further impaired body weight in undernourished children (49). In turn, malnutrition may also affect the immune response through hormonal influences, given the respective reduced and increased leptin and GC levels in undernourished persons (50, 51). In addition to the inhibitory effects of GCs on cell-mediated immune responses (52, 53), leptin also displays immunostimulating effects (54, 55). Leptin deficient animals show atrophy of lymphoid organs, mainly the thymus, which can be reversed upon the leptin administration (56). Accordingly, it may be assumed that the consumption state of TB patients along with the decreased or increased leptin and GCs levels may impact negatively on thymus function.

## Thymus Involvement in TB, Facts, and Hypothesis

Because of the continuous need to replenish mature peripheral T cells that undergo normal turnover throughout life (57), preserved thymus during *M. tuberculosis* infection in the mammalian host may be essential for the development of an effective immune response against mycobacteria.

Animal studies showed that following erogenic infection, the thymus is as likely to be infected with *M. tuberculosis* as the lung tissue (58). Thymic compromise may be observed in bacterial infections, including those caused by mycobacteria, i.e., *M. tuberculosis* and *M. avium* (59–62). Despite some immunological compromise, thymus infection also displays compensatory strategies aimed at improving thymic function; that is the identification of *Mycobacterium-*reactive T cells within the thymus that migrated from the peripheral compartment (63, 64).

As regards to the clinical field, while historical histopathological preparations from old patients identified the occurrence of thymic TB (65) thymic TB is an infrequent presentation of the disease, with a bit more of a dozen cases being reported in the literature (66, 67).

Without being mutually exclusive, it can be assumed that the endocrine abnormalities present in TB may also affect the thymus by mechanisms that go beyond the infection *per se*, resulting equally detrimental, i.e., a deficient immune competence or thymic selection. In normal conditions, bone marrow T-cell progenitors migrate to the thymus to undergo a broad process of differentiation and selection. Thymocyte positive selection is mediated by thymic epithelial cells (TECs), which not only display antigen-presenting activity, but also secrete compounds or express cell surface molecules essential for thymocyte development. In the medulla, medullary TECs allow the T-cell recognition of self-antigens by facilitating the expression of tissue-related antigens and presenting them to developing thymocytes. Central T-cell tolerance also takes place in the thymic medulla, for which the removal of harmful and autoreactive T-cell clones is achieved (68–70). After entering the thymus, thymocytes representing different stages of development occupy distinct regions of the thymus. Thymocyte progenitors referred to as double negative cells (CD3^−^CD4^−^CD8^−^) locate at the cortico-medullary junction, where undergo rapid proliferation, mostly driven by IL-7, and further migrate through the cortex toward the medulla. Cells unable to rearrange their antigen receptor genes will endure apoptosis, whereas those experiencing gene rearrangements of the T-cell receptor genes and acquisition of both CD4 and CD8 coreceptors (CD4^+^CD8^+^ double positive—DP cells) undergo positive (functional TCR) and negative (non self-reactive TCR) selection in the cortex and medulla. Most DP cells have nonfunctional antigen receptors rendering them unable to receive surviving signals for which they undergo apoptosis (death by neglect). The surviving cells, which loss either CD4 or CD8 molecules and become single positive (SP) cells, undergo negative selection; that is an activation-induced cell death of cells with high affinity antigen receptor for self-antigens. Finally, cells leave the thymus as CD4^−^CD8^+^ (cytotoxic) or CD4^+^CD8^−^ (helper), SP mature, naïve T cells (68–70).

Turning to the disturbed immune-endocrine responses seen in TB patients there is reason to believe that such changes, particularly the ones dealing with adrenal steroids and leptin may indirectly compromise thymus function, favoring gland involution. Thymic involution is the progressive loss of the thymus to sustain lymphopoiesis and the ensuing impairment for *de novo* T-cell production. Thymic senescence starts well advanced puberty and by 50 years of age 80% of the thymic stroma is replaced by adipose tissue. The maximum decline in the thymic weight occurs between 30 and 40 years of age (71, 72), which might account for some evidence of a lower thymic activity seen in individuals older than 40–50 years (73, 74).

Besides aging, thymic involution can be provoked by several conditions and factors: among them pregnancy, severe infections, cancer, irradiation and hormones, like GCs (70). In mouse models, high doses of GCs cause thymocyte depletion, involving especially DP thymocytes and TECs (70, 75). Some experimental studies also suggest that GC production at the thymic level may influence thymocyte differentiation and thymic homeostasis (76–78).

According to the neuroendocrine influence on thymic function, infectious diseases and the malnutrition state that may accompany in some cases, i.e., TB, are quite likely to affect thymic activity (79, 80).

Although at the experimental level low GCs concentrations may rescue thymocytes from the TCR-mediated apoptosis (81, 82), the scenario in TB patients is characterized by a chronic elevation of cortisol that while being of moderate intensity remained so even after several months of treatment initiation (83). Furthermore, TB patients also present quite reduced amounts of circulating leptin levels (45). This hormone prevents starvation-induced thymic atrophy (84) along with a protective effect on the loss of lymphoid and TEC populations occurring in the stress-induced acute atrophy of the thymus (85). It follows that increased cortisol and reduced leptin levels promote an unsuitable scenario for a proper thymus function.

**Figure 1:**
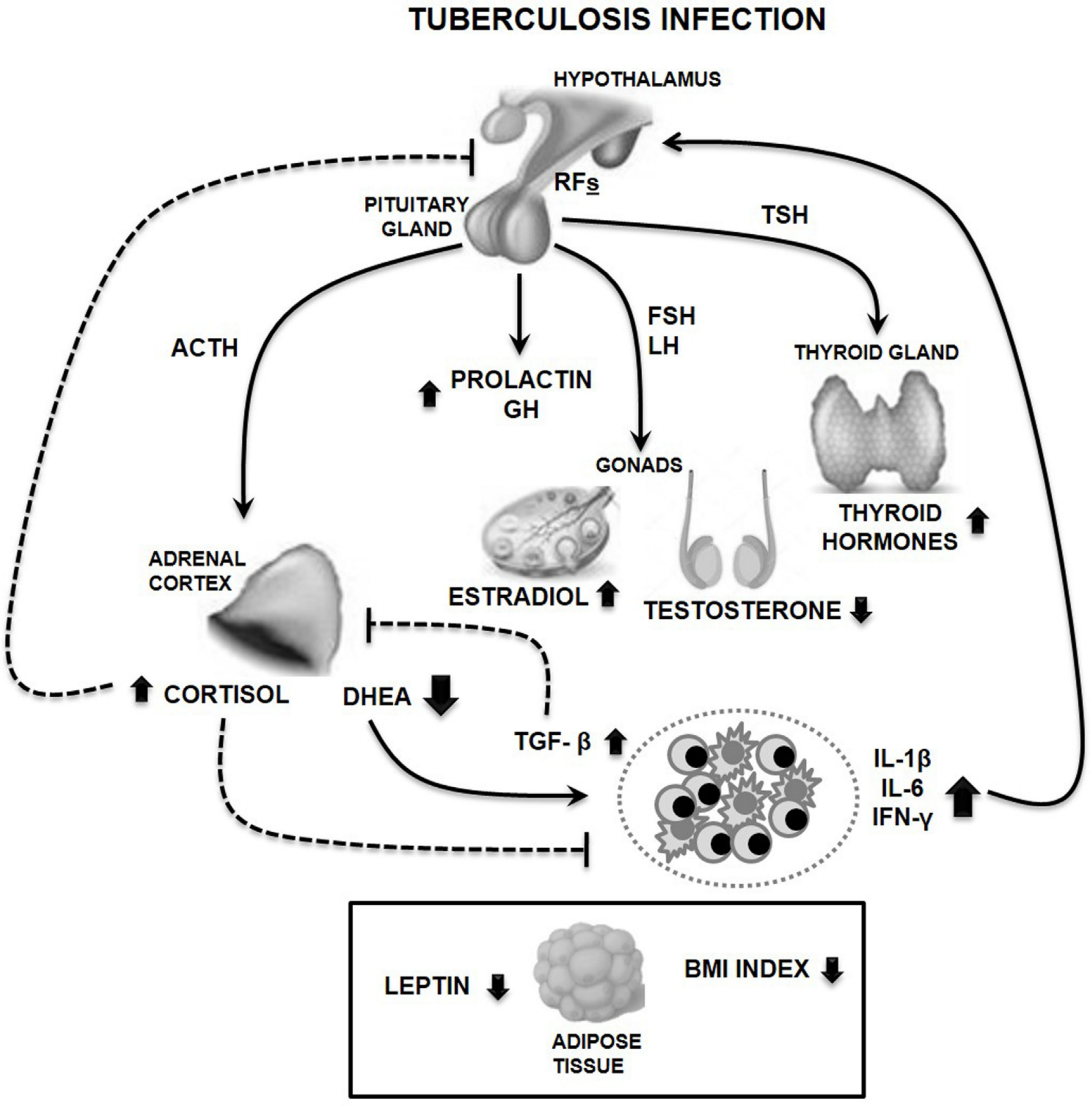
Main features of circulating immune-endocrine alterations in male tuberculosis (TB) patients. Cytokine release by immunocompetent cells stimulates the production of releasing factors (RFs) at the hypothalamic levels, like the corticotropin-releasing hormone leading to the pituitary synthesis of adrenocorticotropin hormone (ACTH). This is followed by the production of adrenal steroids, cortisol, and dehydroepiandrosterone (DHEA), which are, respectively, increased or decreased during TB. Such unbalanced cortisol/DHEA relation along with the altered production of gonadal steroids are much likely to favor a Th1→Th2 immune shift, further accompanied by reduced amounts of leptin, an immunostimulating compound. Presence of transforming growth factor beta (TGF-β) which is increased in TB, in turn, inhibits DHEA production by adrenal cells. TB patients also displayed increased amounts of growth hormone (GH) and prolactin probably related to the protracted inflammation, in addition to augmented levels of thyroid hormones. This pattern of hormonal alterations would favor a deficient infection control together with a catabolic status, as exemplified by the reduced body mass index (BMI) and leptin plasma levels seen in patients (represented in a separate box dealing with a metabolic component). Solid and dashed lines represent stimulating and inhibiting effects, respectively. Abbreviations: FSH, follicle-stimulating hormone; LH, luteinizing hormone; TSH, thyroid-stimulating hormone; IL-6, interleukin 6; IL-1β, interleukin 1 beta; IFN-γ, interferon gamma.

Our study in TB patients showed decreased levels of testosterone and DHEA, in presence of augmented amounts of GH, not accompanied by increased IGF-1 levels, in parallel to modest increases estradiol, prolactin (PRL), and thyroid hormones (30) (a summary of immune-endocrine alterations is provided in Figure 1).

Pretreatment of mice with DHEA was found to result in a partial protection from the GC-induced decrease in thymus weight and thymocyte death (86, 87). Similarly, administration of DHEA to male mice partially or completely reversed the dexamethasone-inhibited blastogenic response to mitogen stimulation (88). Depending on the experimental conditions, *in vitro* treatment with DHEA may promote thymocyte apoptosis (89) or even exert an anti-apoptotic effect on these cells (90). Studies in rats undergoing a repeated immobilization stress showed that DHEA behaved as an anti-stress hormone (91), whereas DHEA supplementation in rats undergoing an experimental *Trypanosoma cruzi* infection led to an improved thymocyte proliferation and reduced TNF-α production (92). Collectively, these findings tip the balance toward a favorable role of DHEA on thymus function, for which reduced levels of DHEA in TB patients may be also disadvantageous. Hormones other than the HPA axis are also likely to influence the thymus gland [reviewed in Ref. (71)]. GH is known to increase the release of cytokines, chemokines and thymulin (93), and to augment the deposition of proteins implicated in cell migration (94, 95); whereas PRL facilitates the survival and proliferation of early T–cell progenitors (96). Aged rat recipients of cells from a pituitary adenoma secreting GH and PRL appeared recovered from the thymic involution (97), as well. The extent to which GH may be operative in our patient series is uncertain since increased GH levels were not accompanied by an increase in IGF-1 implying a state of resistance to GH (30). About PRL, the increase seen in TB patients was quite low as did thyroid hormones (30). In situations of greater exposure thyroid hormones may be beneficial as seen in T3-treated mice (98) or the relation between hyperthyroidism with thymic hyperplasia because of the increased numbers of thymocytes (99). Since the increase in thyroid hormones of TB patients did not fit with a clear hyperthyroidism, we remain unsure on the role of these hormones on the thymic gland.

Some pieces of evidence point out that sex steroid have deleterious effects at the thymic level since thymus atrophy accelerates at puberty (100), whereas administration of androgens or estrogens in adult mice results in a remarkably decreased thymopoiesis linked to an increased apoptosis of cortical thymocytes (101). In our study, testosterone and estradiol were comparatively decreased or increased, respectively (30), for which the thymic role of both steroids in the TB scenario remains uncertain.

Collectively, the evidence discussed indicates a harmful influence of immune-endocrine alterations at the level of the thymus; however, these changes may be reversible and associated with the clinical improvement of patients, leading to an eventual normalization of the thymic function.

The scenario present in TB patients can be conciliated with the view wherein neuroendocrine hormones released in response to psychosocial stress, chronic inflammation or persistent infections are likely to result in premature immunosenescence (102), particularly when considering the resemblance of immune changes seen during aging or chronic GC exposure. In fact, the immunosenescence pattern seen in healthy aging is comparable to the one observed in subjects under chronic stress or chronically exposed to GCs, i.e., thymic involution, declined thymic exportation of naive T cells, a Th1→Th2 cytokine shift, increased circulating levels of pro-inflammatory markers and shorter telomere lengths, compatible with an accelerated aging [reviewed in Ref. (103)].

Notably, senescent cells remain metabolically active for which they may influence other cells through a process termed senescence-associated secretory phenotype (104, 105). That is, the secretions of several inflammatory mediators that exacerbate senescence in the same cell or propagate to the neighbor ones or even systemically amplifying a phenomenon termed inflammaging. Many tissues and cell types participate in producing pro- and anti-inflammatory stimuli dealing with Inflammaging (106). The basis for the establishment of age-related diseases involves an excessive production of pro-inflammatory mediators coupled to an inefficient anti-inflammatory reaction (107). Immunosenescence on its own affects both innate and adaptive immunity, thus providing a contributory mechanism to account for an increased morbidity (108–110).

**Figure 2:**
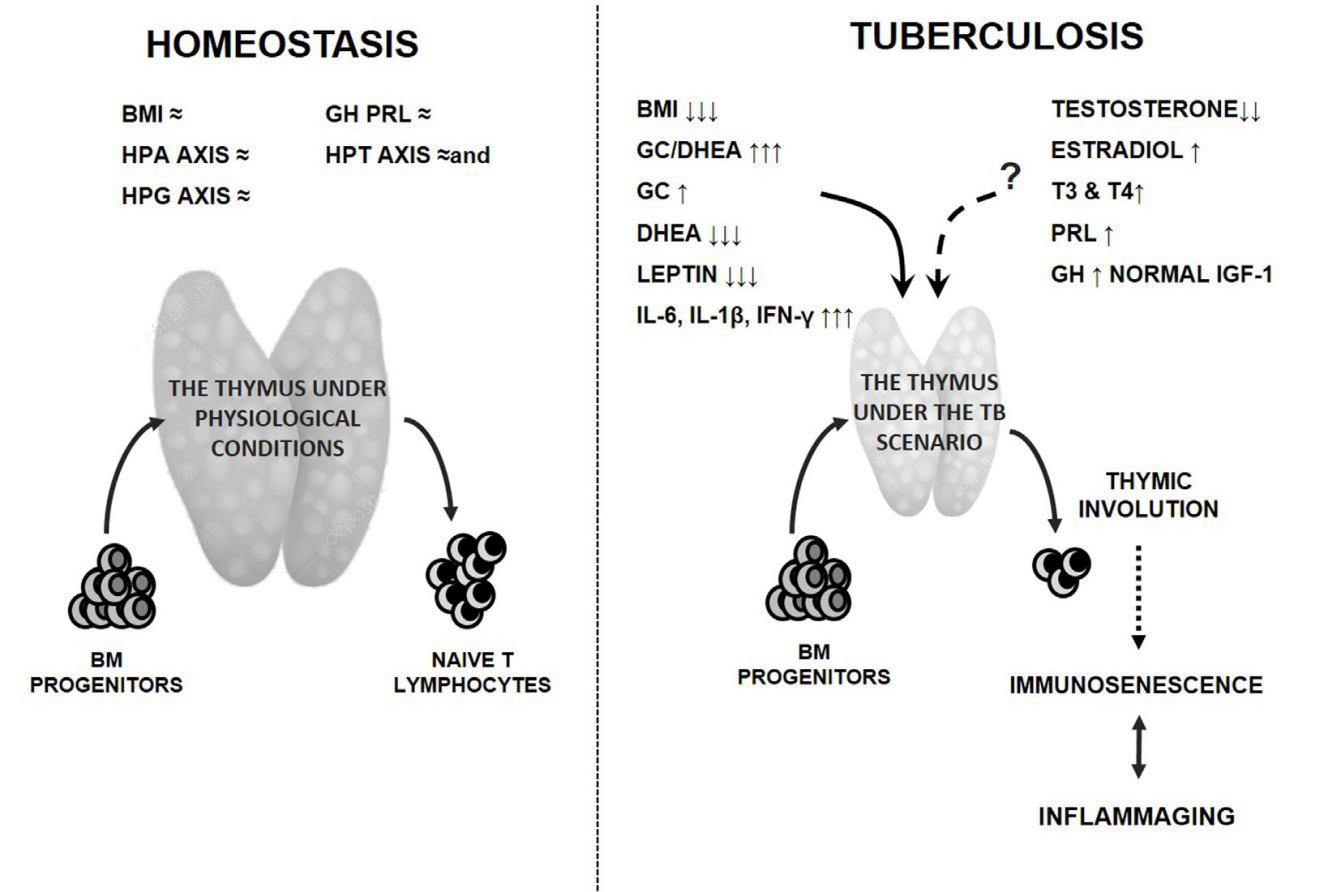
Endocrine alterations in tuberculosis (TB) patients and the potential thymic repercussion. Detrimental effects of clinical and endocrine disturbances on the thymus gland and function during TB are presented by solid lines, which is the consumption state along with the increased amounts of cortisol and pro-inflammatory cytokines in presence of reduced levels of leptin and DHEA. While administration of androgens or estrogens in adult mice leads to a decreased thymopoiesis, the thymic influence of gonadal steroids in TB is uncertain, since patients displayed decreased or increased levels of testosterone and estradiol, respectively (dashed line). Levels of prolactin and thyroid hormones appeared augmented, but their increases did not reach the values able to mediate a clear beneficial effect on the thymus gland (dashed line). The extent to which GH may be favorable at the thymic level remains also unclear since its increased amounts were not accompanied by higher IGF-1 values compatible with state of GH resistance (dashed line). The resulting thymic involution mostly because of leptin and adrenal steroid changes together with a chronic inflammatory state are likely to lead to premature immunosenescence (dotted line) and the coexisting inflammaging. Most of these changes would contribute to worsen the disease course. The left panel represents the preserved (≈) homeostatic situation. Abbreviations: BM, bone marrow; BMI, body mass index; HPA, hypothalamic pituitary adrenal; HPG, hypothalamic pituitary gonadal; HPT, hypothalamic pituitary thyroid axes; GH, growth hormone; PRL, prolactin; GC, glucocorticoids; DHEA, dehydroepiandrosterone; IGF-1, insulin growth factor like 1; T3, triiodothyronine; T4, thyroxine; IL-6, interleukin 6; IL-1β, interleukin 1 beta; IFN-γ, interferon gamma.

A summary of the immune-endocrine alterations encompassing TB and their eventual repercussion on thymic function is provided in Figure 2.

## Concluding Remarks

Tuberculosis is a disease wherein the immune response cannot cope with mycobacteria for which the infection becomes chronic as did the accompanying immuno-inflammatory state. Such situation set the basis for the establishment of an altered immune-endocrine response that will not only impact on the clinical and metabolic status of patients but also on innate and adaptive immune responses. The bulk of evidence discussed here also suggests a still not envisaged view in the sense that immune-endocrine abnormalities, particularly the unbalanced relationship between adrenal steroids along with decreased leptin levels, in a pro-inflammatory milieu, are much likely to impact adversely on thymic function.

Tuberculosis has taught us a great deal in relation to the physiopathogenesis which take place in the context of human infections and chronic inflammation, not least in identifying the complex networks of events underlying the clinical disease manifestation. Despite such successes much remains to be accomplished. Importantly future studies are needed to appraise the extent of thymic affectation during active disease, the eventual repercussion on the immunological status of patients, mainly in the context of progressive disease, multidrug-resistant TB, or HIV coinfection. An elucidation of these novel pathogenic avenues will lead ultimately to the development of better diagnostic or therapeutic tools facilitating a more integral strategy for disease control.

## Author Contributions

LD, NS, BB, MB, and OB conceived, designed, and performed the studies serving to prepare the review. LD, NS, BB, MB, and OB wrote the paper.

## Conflict of Interest Statement

Authors declare that the research was conducted in the absence of any commercial or financial relationships that could be construed as a potential conflict of interest.
